# Low-Intensity Self-Management Intervention for Persons With Type 2 Diabetes Using a Mobile Phone-Based Diabetes Diary, With and Without Health Counseling and Motivational Interviewing: Protocol for a Randomized Controlled Trial

**DOI:** 10.2196/resprot.2768

**Published:** 2013-08-26

**Authors:** Lis Ribu, Heidi Holmen, Astrid Torbjørnsen, Astrid Klopstad Wahl, Astrid Grøttland, Milada Cvancarova Småstuen, Elisabeth Elind, Trine Strand Bergmo, Elin Breivik, Eirik Årsand

**Affiliations:** ^1^Department of NursingFaculty of Health SciencesOslo and Akershus University College of Applied SciencesOsloNorway; ^2^Department of Health Sciences, Institute of Health and SocietyFaculty of MedicineUniversity of OsloOsloNorway; ^3^Norwegian Centre for Integrated Care and Telemedicine (NST)University Hospital of North NorwayTromsøNorway; ^4^Institute of Clinical MedicineFaculty of MedicineUniversity of OsloOsloNorway

**Keywords:** self-management, empowerment, health-related quality of life, acceptability, type 2 diabetes, lifestyle intervention, complex intervention, mHealth, telemedicine, motivation, health counseling, mixed methods

## Abstract

**Background:**

The present study protocol is designed to cover the Norwegian part of the European Union Collaborative Project—REgioNs of Europe WorkINg together for HEALTH (RENEWING HEALTH). Self-management support is an important element of care for persons with type 2 diabetes (T2D) for achieving metabolic control and positive lifestyle changes. Telemedicine (TM) with or without health counseling may become an important technological aid for self-management and may provide a user-centered model of care. In spite of many earlier studies on TM, there remains a lack of consensus in research findings about the effect of TM interventions.

**Objective:**

The aim of RENEWING HEALTH is to validate and evaluate innovative TM tools on a large scale through a common evaluation, making it easier for decision makers to choose the most efficient and cost-effective technological interventions. The Norwegian pilot study evaluates whether the introduction of a mobile phone with a diabetes diary application together with health counseling intervention produces benefits in terms of the desired outcomes, as reflected in the hemoglobin A1c level, health-related quality of life, behavior change, and cost-effectiveness.

**Methods:**

The present study has a mixed-method design comprising a three-armed prospective randomized controlled trial and qualitative interviews with study data collected at three time points: baseline, after 4 months, and after 1 year. The patients’ registrations on the application are recorded continuously and are sent securely to a server.

**Results:**

The inclusion of patients started in March 2011, and 100% of the planned sample size is included (N=151). Of all the participants, 26/151 patients (17.2%) are lost to follow-up by now, and 11/151 patients (7.3%) are still in the trial. Results of the study protocol will be presented in 2014.

**Conclusions:**

The key goals of this trial are to investigate the effect of an electronic diabetes diary app with and without health counseling, and to determine whether health counseling is important to the continued use of the application and the patients’ health competence and acceptability. Research within this area is needed because few studies have investigated the effectiveness of apps used in long-term interventions with this degree of self-management.

**Trial Registration:**

Clinicaltrials.gov NCT01315756; http://clinicaltrials.gov/ct2/show/NCT01315756 (Archived by WebCite at http://www.webcitation/6BTyuRMpH).

## Introduction

### Overview

The prevalence of type 2 diabetes (T2D) is increasing throughout the global population [1]. A similar trend is also evident in the Norwegian population, where 192,421 people of the total population (5,063,709) are estimated to have diabetes, with a prevalence of 3.8% [2]. The impact of T2D is serious for both the individual and society with considerable economic costs [1,3]. Although medical competence for treating diabetes is improving and knowledge about treatment and lifestyle factors relating to T2D is substantial, most people with this disease do not achieve metabolic control [4,5], and can therefore experience diabetic complications [6].

The recent Coordination Reform in Norway has reorganized the distribution of health resources with an increased emphasis on the development of services within municipalities [7]. According to the Norwegian guidelines for diabetes, this organizational redirection complies with the current recommendations for the treatment of patients with T2D [8]. Earlier research in Norway indicated a gap between the guidelines and current clinical practice because only one of eight patients reaches the combined goal of control of glycemia, blood pressure, and lipids [9]. However, improvements in primary care for patients with T2D have been observed in Norway in recent years [10].

A systematic review has shown that diabetes self-management education for adults with T2D is effective when delivered in a community context [11]. Lifestyle changes such as increased physical activity and improved dietary habits may influence and improve metabolic control in persons with T2D [12]. To succeed in achieving positive lifestyle changes, the patient must be involved, and self-management support is an important element in the care of persons with diabetes [13] as well as persons with any chronic diseases [14]. Enhancing diabetes self-management strategies has shown promising results for reducing hemoglobin A1c (HbA1c) level in this group, a change that plays an important role in both reducing the risk for developing complications and improving the quality of life [15]. Nurse-delivered combined motivational enhancement therapy and cognitive behavior therapy has been shown to be feasible for adults with poorly controlled type 1 diabetes and can lead to an improvement in HbA1c levels [16]. However, further research is needed for optimizing health outcomes in these settings and for learning how to design more individualized approaches.

The psychological burden on the individual patient caused by the disease must also be recognized. Diabetes care providers such as nurses and physicians must deal with patients’ everyday problems in managing diabetes, and some patients may need to seek help from psychosocial specialists [17]. Some earlier research have shown a high prevalence of depression in persons with T2D and an association between depression and poor self-management, poor metabolic control, and diabetic complications [18-20].

When living with and managing a chronic disease at home, telemedicine (TM) has the potential to become an important aid for self-management and may also help ensure a user-centered rather than a biomedically centered model of care [21]. Modern technology combined with psychological interventions can be useful in providing efficient management of diabetes. The TECNOB study is an example of how to use technology and a cognitive behavioral approach in a multidisciplinary telecare intervention for weight loss in obese patients with T2D in inpatient treatment (1 month) and in the continuity of care at home (1 year) [22,23]. Interactive systems that integrate monitoring and personalized feedback functions should be developed [24]. The importance of clarifying the effect of interventions that combine telemonitoring with educational and motivational tools, and those consisting of telemonitoring only, is also of interest [25]. Evaluations of TM interventions for persons with diabetes have mainly focused on the achievement of a clinical outcome in terms of glycemic control [26,27] with a reported trend toward patients achieving better glycemic control [28,29]. Few studies have found improvements in participants’ quality of life [24,28-30], but one review investigating the impact of home telehealth interventions on the patients’ quality of life and patient satisfaction compared with usual care [29] refers to a study with a single-group design that indicates significant improvements in physical functioning, bodily pain, and social functioning after 1 year of home telehealth [31]. Satisfaction with the new technologies has also been demonstrated, and more complex interventions with definitions of the process of care, and links between patients and professionals, showed better outcomes [30]. It is important to know whether users accept this type of service and the term “acceptability” is often used to indicate the degree to which patients are satisfied with a service and are willing to use it [32]. It is, therefore, recommended to design studies considering the patients’ need for technology support [24]. Although diabetes telemonitoring has been shown as an effective approach both for glycemic control and for self-management, more research within this area is needed. Systematic reviews indicate that TM systems can be used effectively for persons with diabetes, although this conclusion is based on weak evidence. Further research should seek to understand how TM may improve diabetes management and enhance educational and self-management interventions [33,34]. It has, for example, been shown a gap between the evidence-based recommendations and the functionality of the application features used in study interventions for diabetes care [33]. Despite a large number of studies on TM and systematic reviews on the effects of TM, a systematic review of existing reviews raises questions about the quality of the research evidence in terms of the approaches to evaluation and the methodologies used. The authors indicated the need for more focus on patients’ perspectives, economic analyses, and TM innovations as complex processes and ongoing collaborative achievements. Formative assessments are also of interest [35]. There is a need for studies to be designed to control for possible mediating variables [28]. It has earlier been shown how positive clinical outcomes might be associated with mediating variables (or process variables) such as intensified provider consultation [36], more active management [37], or cognitive processes [38].

### Aim of the Study

The present study is the Norwegian part of the European Union (EU) Collaborative Project—REgioNs of Europe WorkINg together for HEALTH (RENEWING HEALTH). The aim of this study is to validate and evaluate innovative TM tools on a large scale using a common evaluation method, thereby making it easier for decision makers to choose the most efficient and cost-effective technological aids [39]. The Norwegian study evaluates whether the introduction of personalized and technology-supported self-management with and without health counseling intervention produces benefits in desired outcomes, as reflected in HbA1c level, health-related quality of life, behavior change, and cost-effectiveness.

### Theoretical Framework

In the present study, we perform a self-management intervention focusing on behavior change and the implementation of the evidence-based approach to diabetes self-management education and self-management support [13]. Self-management has been described as “the nature and scope of the ways in which patients state that they need to change to become more active participants in maintaining their health” [14], and the World Health Organization “white paper” has described self-management as a set of cognitive and behavioral self-management skills such as coping skills, goal setting, self-monitoring, environmental modification, self-reward, and arranging social support [40]. The health counseling part of the present study is based on principles from cognitive behavioral therapy, the “Reach Out” problem-solving model [41,42], and motivational interviewing (MI) [43]. The diabetes specialist nurse will use principles of MI such as a person-to-person interview with a client-centered style for eliciting behavior change by helping a patient explore and resolve ambivalence. MI is a refined form of the familiar process of guiding and is a technique that complements the communication skills needed by nurses and other health care workers [43]. The transtheoretical model that provides the diabetes specialist nurse a way of grouping the patients according to their stage of readiness to adopt new behaviors is also used [44,45]. Patients with T2D may have a wide variety of problem behaviors related to diet, physical activity, medication, and smoking. The transtheoretical model focuses on the patient’s readiness to change and on the individual’s decision making. The model has been used earlier to identify patients with diabetes at different stages of readiness to change to a healthy diet [46]. It has also been used in a successful telephonic intervention to improve diabetes control in urban adults in which patients were grouped according to their stage of change within different lifestyle domains [47]. The diabetes specialist nurse has a role as supporter, helping motivate the patients, although the real work is being undertaken by the patients themselves [41,42]. The term “low-intensity” (intervention) can be seen as a “lower dose” of specific treatment technique that may represent less support from the health personnel (the diabetes specialist nurse in our study) in duration and frequency of contact, and is usually delivered in a nontraditional way such as by mobile phone [41,42].

Glasgow et al [48] documented that it is necessary to include patient-reported psychosocial and behavioral measures in research to address the need for support in persons with diabetes.

## Methods

### Study Design

The study has a mixed-method design comprising a three-armed prospective randomized controlled trial (RCT) and qualitative interviews. The study has been registered with Clinical Trials (NCT01315756). Mixed-method research is a purposeful combination of quantitative and qualitative methods to enrich the material and to obtain a broader understanding of the findings in a study [49]. There are different ways to conduct the investigation and different ways of positioning the involved methods in relation to each other. In our study, the primary outcome measure is based on the findings from the quantitative part, and the qualitative interviews were based on grounded theory to provide us with additional information about the accessibility of the study and the intervention process.

**Figure 1:**
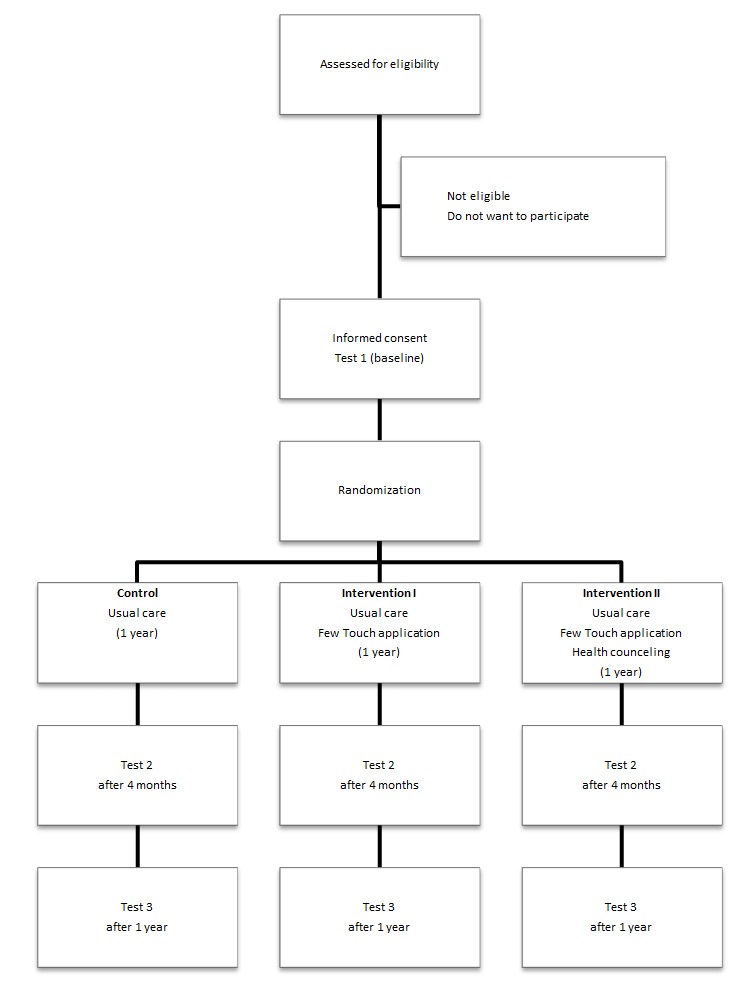
Design of the randomized controlled trial.

The present study has a longitudinal design, and study data are collected at three time points: Test 1, at the time of inclusion (baseline); Test 2, after 4 months; and Test 3, at the end of the 1-year study. The patients’ registrations on the diabetes diary app, called the Few Touch application, are recorded continuously and are transferred securely to a server. The participants in the two intervention groups are using the TM application during the 1-year study (Figure 1); one of these groups additionally receives health counseling during the first 4 months.

### Trial Population and Recruitment

Persons with T2D are eligible to participate in the study if they are older than 18 years, were diagnosed with diabetes for more than 3 months before the study inclusion, and have an HbA1c level >7.0%. Patients must be cognitively able to participate, understand, and be able to complete questionnaires in Norwegian language, and be able to use the mobile self-management system provided to the intervention groups. The exclusion criteria are any mental or physical conditions that interfere with the protocol.

Patients treated in primary care from both the southern and northern parts of Norway are recruited by their general practitioners (GPs). The GPs obtain recruitment information from the research team and are asked to recruit eligible patients to the study. Patients are also recruited from “diabetes start courses” held by the specialist health care service, which are offered to those newly diagnosed with diabetes in Norway by local public health clinics in municipalities and by advertisement. Patients willing to participate in the study are first given a letter with a brief summary of the study and an invitation to obtain more in-depth information about the study and implications. This information is given both orally and in written form in start-up meetings arranged by the project team. Before entering the study, all patients are required to provide written informed consent about their participation. All patients included must be under the care of a GP who adheres to national guidelines for diabetes care.

### Randomization

Patients who meet our inclusion criteria sign the informed consent form, complete the self-reported questionnaire, and are randomized into one of the three groups through block-randomization in the start-up meeting described above. Randomization is performed through the Center of Randomization at the Unit for Applied Clinical Research at the Norwegian University of Science and Technology in Trondheim, using WebCRF (Case Report Form). Immediately after randomization, the patients are told which group they have been placed. Those placed in the intervention groups are given a mobile phone with the self-management application in the same meeting and taught how to use the phone and the application. Those randomized to both the application and the health counseling group is given information about this intervention at the end of the same meeting. The reason for choosing this procedure is to save time for those who travel a considerable distance to participate.

### Development of the Intervention

#### Overview

All participants receive usual care by their GP. The patients in the control group receive only usual care. Usual care in Norway is regulated by national guidelines, which include at least one annual visit to a GP. Standard measurements are blood pressure, serum concentrations of lipids and glucose, HbA1c level, and weight and body mass index. The patient’s regular visit with a GP includes treatment for elevated blood glucose, blood pressure, and lipids when needed. The GP also emphasizes the importance of lifestyle changes [8].

There are two intervention groups (I+II) in our study. In addition to usual care, the participants in both the intervention groups receive a mobile phone with the diabetes diary app referred to as the Few Touch application, which is a self-help tool comprising five elements that are accessible to the user: (1) food habits registration system, (2) blood glucose data management system, (3) physical activity registration system, (4) personal goal-setting system, and (5) general information system. Blood glucose data are transferred automatically to the mobile phone-based diabetes diary from the blood glucose meter when the user has performed a measurement. Activity data and food habits are entered manually by the user. The users can also set personal goals for physical activity and food habits, access related tips, and look up words and concepts related to their disease.

In addition to the mobile phone, intervention group II also receives theory-based health counseling delivered by a diabetes specialist nurse working at a diabetes outpatient clinic at a university hospital, and with the possibility of support from a dietitian. The health counseling intervention comprises five modules (Table 1) and is delivered over 4 months immediately following the randomization. The nurse sends standardized short messages (using short message service, SMS) a few days before calling to inform the patients of the planned content in the phone conversation. The patients are also able to initiate SMS text messaging to communicate with the diabetes specialist nurse.

**Table 1:** Five modules of the health counseling intervention.

Module	Module theme
Module 1 (intro)	Introduction
Module 2 (1st month)	Living with diabetes
Module 3 (2nd month)	Goal setting
Module 4 (3rd month)	Diet and physical activity
Module 5 (4th month)	Looking back and continuing forward

#### The Mobile Self-Management Tool

Several projects for designing various mobile self-management systems within diabetes have been conducted at the Norwegian Centre for Integrated Care and Telemedicine in Norway. The T2D tool, the Few Touch application, has been developed together with and tested on 12 patients with diabetes, as described in a previous publication [50]. The app was both designed and tested in close contact with persons with diabetes over a 3-year period. Obtaining constant feedback from participants and having the ability to change the app as needed during the testing-period, helped in making it a user-friendly and practical app. Initial promising results were demonstrated for this user-involved design process involving people with T2D [51].

#### Health Counseling

The health counseling intervention given to intervention group II is based on a problem-solving method and is delivered by the nurse through short telephone conversations with the patients. The diabetes specialist nurse supports the patients to (1) assess problems, (2) identify possible solutions, (3) analyze strengths and weaknesses, and the main advantages and disadvantages of each solution, (4) select a solution based on the analyses in the stage 3, (5) plan implementation, (6) implement the solutions, and (7) review the process and outcome. This problem-solving model is an evidence-based low-intensity intervention with a practical and systematic approach. It was developed for counseling and guided self-help for groups of patients in mental health services in the United Kingdom. The model has been described as useful for patients with diabetes and other chronic diseases, and requires less support from the health care provider in terms of duration and frequency of contact [41,42].

One of the aims of the present study is to activate the patients’ motivation toward self-management. In this regard, the MI technique includes the following approaches in the conversation between the patient and the clinician (the diabetes specialist nurse in our study): (1) establishing a collaborative partnership between the patient and the clinician to find a solution, (2) evoking from the patients what they already have in terms of their own motivation and resources for change, and (3) honoring the patients’ autonomy about how to live their lives. The clinician has to resist the “righting reflex,” understand the patients’ motivation, and listen to and empower the patients [43].

**Figure 2:**
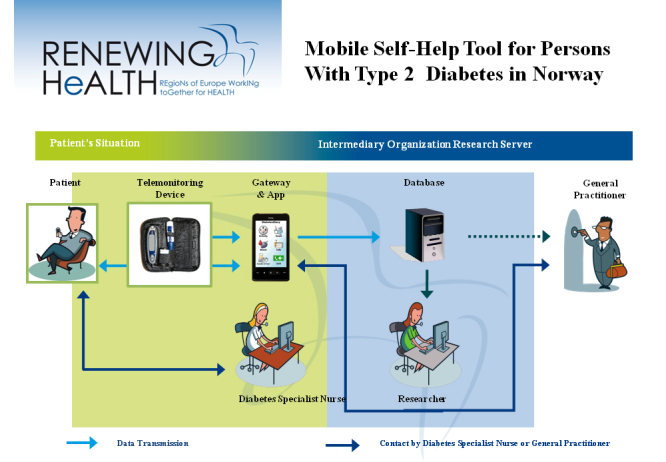
Pilot drawings.

To guide the participants in the change process, the nurse assesses the motivational stages [44] for each patient in relation to diabetes-relevant areas and the use of the Few Touch application. The health counseling is based on national guidelines [8], and the intervention as illustrated in Figure 2 is explained in the following text.

At the first stage, patients set goals for what kind of food intake and physical exercise they plan in the next period. Then, the blood glucose level is measured with Bluetooth-enabled glucose meter that transfers blood sugar level data automatically to the mobile phone. Thereafter, patients add food intake and activity manually on the phone application. Accordingly, patients get response from the application on how the individually set goals are met within the defined period.

Parallel to the registration process, the following activities are also carried out. First, patients in the health counseling group have contact with the diabetes specialist nurse in parallel to using the application, where relevant health-related questions and results are discussed. Second, patients in both the intervention groups (I+II), have regular consultations with their physician where they will be able to share their application data, if they choose to do so. Finally, responsibility and control of their data lie with the patients.

#### Information and Training

Both intervention groups (I+II) are instructed in using the mobile phone-based system. In the first session, they are given a practical presentation of the diabetes diary from the project group, which describes the different details recorded in the diary. This is followed by a session in which the patients try the same functionalities on their mobile phone. They are also given a manual for the use of the mobile phone and the Few Touch application both in a paper-based handbook and on a USB memory stick. The telephone support service is available on weekdays from 9:00 to 15.00 h, when support providers are available to answer questions and help the participants with technical aspects. The patients are also informed through the consent form that the project team might contact them if they are not using the phone at all, which is discovered by lack of registrations on the central database server. Participants in intervention group II who additionally receive the health counseling are given additional training in how to send and receive SMS messages securely to the diabetes specialist nurse or dietitian.

#### Power Analyses

Power analyses were performed before recruitment to estimate the sample size needed based on the HbA1c level as the primary outcome. Given an effect size of 0.35, a significance level of 5%, a standard deviation of the outcome variable of 0.5, statistical power of 80%, and a two-tailed significance test, the sample size was estimated to be 34 individuals in each group. To compensate for dropouts, the sample was set to 50+50 (two intervention groups), and 50 in the control group. A total of 151 patients were included.

### Evaluation Measures

#### Overview

Our evaluation is based on the Model for Assessment of Telemedicine (MAST), which is a framework developed for the evaluation of the TM interventions in RENEWING HEALTH [39]. We are also using principles from the complex intervention framework developed by the Medical Research Council in the United Kingdom [52]. We intend to evaluate a large number and variety of outcomes. The consolidated standards of reporting trials (CONSORT) statements for reporting parallel group randomized trials are followed [53,54]. The study data include responses on self-reported questionnaires, logs from the Few Touch application, and data collected from patient journals obtained from the GPs. To assess the representativeness of the sample, we will use data from the general population with and without diabetes from the Nord-Trøndelag Health Study (HUNT 3) in Norway [55]. We will also conduct in-depth interviews with patients and health care providers consecutively. In addition, we will collect data from the technical support service to get knowledge about who are using the service, frequency of use, and what they are asking for. Further, we collect notes from the diabetes nurse taken during the health counseling. The measures in our study are described in detail below.

#### Demographics

These are self-reported measures and include age, sex, education, cohabitation, marital status, and working status of the participant.

#### Clinical Measures

Data from the medical records include medication, dosage, treatment period, and adjustments during the study period. Data about height, weight, blood pressure, and waist circumference are also obtained from medical records. Biological data include HbA1c and lipid levels. Frequency of hypoglycemia and comorbidities are self-reported, and late complications such as atrial fibrillation, intermittent claudication, cerebrovascular disease, coronary disease, and microalbuminuria are obtained from the GPs.

### Primary Outcome Measure

The primary outcome is the difference between the control group and the intervention groups regarding change in HbA1c level after 4 months and after 1 year. When possible, HbA1c levels are recorded within 2 weeks before or after the expected date of follow-up (ie, within a 1-month window) [56]. HbA1c level has been shown to vary between 100 laboratories in the United Kingdom and between the various HbA1c assays in use [57]. In the present study, patients are included from different GP offices, and there may be variations in HbA1c levels recorded. To avoid bias potentially introduced through the use of different HbA1c assays, the DCA Vantage Analyzer from Siemens is used when possible.

### Secondary Outcome Measures

#### Short Form-36 for Measuring Health-Related Quality of Life

This questionnaire contains eight conceptual domains within physical functioning and mental health giving scores of 0-100: (1) physical functioning, (2) role-physical, (3) bodily pain, (4) general health, (5) vitality, (6) social functioning, (7) role-emotional, and (8) mental health [58,59]. It is one of the most frequently applied surveys for measuring health-related quality of life, and its domains are relevant for people with diabetes [60]. The Short Form-36 (SF-36) was included in RENEWING HEALTH to allow comparisons between different regions included in the project and to allow comparisons with norms.

#### Center for Epidemiological Studies Depression Scale for Measuring Depression

The Center for Epidemiological Studies Depression Scale is a tool for measuring depressive symptoms, but not for diagnosing depression [61]. It assesses the behavioral, cognitive, and affective symptoms of depression, and measures sleep disturbance and loss of appetite, which can be important signs of depression and may have major impact on diabetes management.

### Behavior Change and Empowerment

#### Health Education Impact Questionnaire (heiQ)

The Health Education Impact Questionnaire (heiQ) contains 40 questions to measure the effectiveness of patient education and self-management interventions for people with chronic conditions, as well as the patients’ health competence. It is an eight-scale questionnaire with the following domains: (1) positive and active engagement in life, (2) health-directed behavior, (3) skill and technique acquisition, (4) constructive attitudes and approaches, (5) self-monitoring and insight, (6) health service navigation, (7) social integration and support, and (8) emotional well-being. The instrument has been proven to be valid and reliable [62]. The instrument has recently been translated to Norwegian, and a psychometrically testing is ongoing.

#### Diabetes Empowerment Scale-Short Form

The Diabetes Empowerment Scale-Short Form (DES-SF) has eight items related to managing the psychosocial aspects of diabetes, and measures readiness to change and achieve goals [63,64]. The DES-SF has been proven to be valid and reliable, although the short form has not been through a test-retest validation [65]. This instrument has been translated into Norwegian for the present study.

#### Physical Activity

Measures of physical activity are collected by the same questions as in the HUNT 3 study for comparison purposes with a diabetes sample from the general population. We have also included one question based on the transtheoretical stages of change to assess the respondents’ motivation toward physical activity and change in activity level [66].

#### Nutritional Habits

Measures of nutrition are collected using the same questions as those used by the Cancer Registry of Norway in the Norwegian Colorectal Cancer Prevention study, which collects data about the intake of fruit, vegetables, and fat [67]. Motivation for changing nutritional habits is assessed using a question based on the transtheoretical stages of change [46].

### Patient Acceptability

#### Measurement of the Usability of the Mobile Self-Management Tool

The usability of the mobile self-management tool FTA Touch application will be measured by the System Usability Scale (SUS) that comprises 10 items to provide a global view of the patients’ subjective assessment of usability. The SUS has been found to be a “valuable and robust tool in helping assess the quality of a broad spectrum of user interfaces” [68]. The measure has been used earlier in Norway [50].

#### Assessment of the Patients’ Perceptions

To assess the patients’ perceptions about the mobile self-management tool Few Touch application, the patient acceptance questionnaire, Service User Technology Acceptability Questionnaire, used in the Whole Systems Demonstrator program [69] is used in all pilot studies of RENEWING HEALTH. This questionnaire contains 22 items within the following domains: utility of the kit, effect on health status, effects on access to care, effects on health care/social care, privacy, suitability of the kit, and satisfaction with the kit.

#### Patients’ Experiences With the Intervention

We are also integrating a qualitative evaluation with in-depth interviews of consecutive patients at the end of the study. We are using grounded theory [70] and have developed a semistructured interview guide to obtain patients’ experiences and their degree of satisfaction with the intervention. Integrating a qualitative approach into this RCT may strengthen the description of its effects. The interviews will be conducted by a researcher who had no earlier contact with the patients. The interviews will continue until saturation of data is achieved.

### Health Care Perspectives

Health Care perspectives will be collected through semistructured interviews with health care professionals, doctors, and nurses in the GP offices and the diabetes outpatient clinics involved in this study. The focus for this evaluation will be facilitators for and obstacles to using the system provided or similar systems.

### Cost-Effectiveness

The objective of the economic study is to analyze whether the interventions in addition to standard care are cost effective compared to standard care alone. The study will be conducted from a societal perspective; all costs and consequences falling on the health service as well as on the patient and their employers will be included. The analysis will include data on investments in the TM app, as well as training costs, cost of maintenance, and technical support. Data on health resource use (such as GP visits, hospital admissions, and contacts with nurses), transportation costs, and costs of foregone production are also collected. Market prices, tariffs, and average wages (including employment costs) will be used as cost weights or unit costs and will be presented alongside resource data. Total costs of the treatment arms will be compared with the benefits of improving the patients’ health. Health benefits will be measured in quality-adjusted life years (QALYs). Patient-specific QALYs will be computed from the SF-36 Health Survey by employing an estimated preference-based algorithm developed by Brazier et al [71]. QALYs will be calculated by using under the curve analysis, with linear interpolations between utility scores collected at baseline, 4 months, and 12 months assessments. Incremental cost per QALY will then be calculated and incremental cost-effectiveness ratios will be reported and presented on the cost-effectiveness plane [72].

### Statistical Analysis

Baseline measurements will be presented using descriptive statistics. The participants will be block randomized, and the groups should therefore be equal in age and gender distribution. The groups will be compared with regard to clinically relevant variables to ensure they are comparable. Because of the limited size of the groups, they may differ on some variables and, when such variables are identified, they will be adjusted for in the statistical analyses, for example, by treating them as possible confounders.

Baseline data will be compared with data from the normal population (HUNT 3 study) to assess whether our sample is representative of the entire population of Norwegian individuals living with diabetes.

The differences in the main outcome between baseline and 4 months and between baseline and 1 year will be compared between the three groups. First, the crude difference will be analyzed using analysis of variance with post hoc tests (differences between groups). The difference between baseline and the following two measurements within each group will be analyzed using a *t* test. When a change in a selected outcome variable is identified, this change will be modeled using linear regression adjusted for possible confounders.

The cost-effectiveness data will be analyzed using standard statistical analyses depending on the distribution of the data. Missing values will be replaced by imputation. Mean values and 95% CI will be reported for each component of resource use as well as for total costs and effectiveness. Sensitivity analyses will be conducted to handle nonsampling variation.

Our study has a longitudinal design and we expect some dropouts. Therefore, we will perform two analyses: one based on intention to treat and another based on the participants who actually participated actively in our study (per protocol).

### Ethical Considerations

The study has been approved by the Regional Ethics Committee. The patients will be guaranteed full confidentiality and are required to provide informed consent to participate in the study. The security associated with the server will ensure the safety of the data. Only anonymous data are sent in an encrypted way over the Internet, and the data will be stored anonymously. There are ethical concerns about clinical trials when some of the patients receive the intervention, while others do not. However, all patients will receive the usual care by their GPs, who will be aware of their patients’ participation in the study.

## Results

The inclusion of patients started in March 2011, and 100% of the planned sample size is included (N=151). Of all the participants, 26/151 patients (17.2%) are lost to follow-up by now, and 11/151 patients (7.3%) are still in the trial. Results of the study protocol will be presented in 2014. The aim of this study is to validate and evaluate innovative TM tools on a large scale through a common evaluation, making it easier for decision makers to choose the most efficient and cost-effective technological interventions.

## Discussion

Despite a large number of studies of TM and systematic reviews of the effects of TM, there remains a lack of consensus in research findings [28]. The key elements in this trial are the effect of an intervention that combines a mobile self-management tool with motivational support and the comparison with an intervention comprising TM alone. It is of interest to investigate the effect of the TM application with and without health counseling, and it is reasonable to question whether health counseling is important for the continued use of tools such as those in this study and for patients’ health competence and acceptability. One might question whether the health counseling part of the intervention will motivate the patients or whether the repeated phone calls from the diabetes nurse will become more tiresome than supportive and thus unwanted. Research on this question is needed, but to our knowledge, no studies have investigated this thoroughly.

We have chosen to include adult patients of all ages and some not familiar with mobile phones. Our qualitative approach will allow us to search for possible limitations in the use among different groups of patients. This may give us valuable knowledge about how to encourage the use among different groups of patients and how to assess the effects of a low-intensity intervention on different subgroups. Our qualitative approach might also reveal important information in discussions with patients and health personnel about how to integrate self-management tools in the health care system.

The patients included in the study may represent those who are open to new technology and who are willing to participate in a complex intervention; thus, the acceptability to using such devices may be lower in the wider population. It will be important to assess representativeness. In Norway, we have data from large population-based studies that are well suited for comparison purposes. Recruitment to RCTs is also frequently problematic [73,74], and one concern in the present RCT is whether patients who are eligible will be disappointed when they are randomized to the control group and not to an intervention group receiving the Few Touch application or the application together with health counseling. There is reason to question whether an RCT is suited to this type of study when we are recruiting possibly motivated patients to participate in a TM intervention and they may be randomized to the control group. Different designs that are more flexible and clinically useful have been discussed [73], and our study may contribute to this discussion, which is of current interest.

In the present study, we have chosen HbA1c level as the primary outcome. Many people with diabetes have poor understanding of their HbA1c level, and only few remember their actual test results. The level of agreement between perceived glycemic control and actual HbA1c values is also poor [75]. This could be a challenge in our research when the primary outcome is a measure of which many patients may have a poor awareness and understanding. In addition, glycemic control may not be a goal for some patients when they volunteer for the study. However, the patients in the intervention groups will have access to information about their blood glucose measures through the Few Touch application, and one of the intervention groups will additionally receive health counseling to help them attain self-management and diabetes control.

Evaluations of telehealth interventions for persons with diabetes have focused mostly on the achievement of a clinical outcome in terms of glycemic control. Nolte et al [38] performed an in-depth analysis of outcome measures used to evaluate chronic disease self-management programs and found that decisions about the value and efficacy of chronic disease self-management programs should be interpreted with care. There is an important difference between clinical measures (eg, HbA1c level) and patient-reported outcomes. The former can be assessed relatively accurately, whereas assessments of patient-reported outcomes have considerably more measurement error and varying degrees of bias caused by question interpretation and personal appraisal. There is a need for further research, qualitative studies in particular, to unravel the cognitive processes and the role of response shift bias in the measurement of change [76]. We will take into account this challenge in our study by using several evaluation-based measures requiring a large amount of personal judgment. Our mixed-method design and inclusion of in-depth qualitative interviews of consecutive patients leaving the study will help us identify their experiences and reflections, and will provide a more comprehensive description of the effects of processes in our evaluation of the intervention.

Another concern in a complex intervention such as ours is the benefit of the attention that all the participants receive during the study period from different persons, for example, the effect from the patients’ awareness that they are participants under study (Hawthorne effect) [77]. The intervention groups receive attention from the technical support team during the 1-year use of the tools provided, and all groups may receive attention during the collection of data at the three time points. Finally, the GPs will be aware of “their” patients’ participation in a study in which HbA1c is the primary outcome, and this may induce them to provide better treatment. We must avoid the effects of other possible interventions given by these professionals. The possibility that improvements may be caused by intensified provider consultation has been described in a recently conducted review [28], and researchers should be aware of this problem.

Research on barriers to intervention has been requested within diabetes self-management interventions [78] and health research in primary care [79]. Barriers to participation and adoption of TM from the perspective of people who decline to participate or who withdrew early from the trial have been described [80]. We do not have permission from the ethical committee to ask patients who drop out during the study for the reasons. In-depth interviews at the end of the study will, however, give us qualitative data about the patients’ views of the intervention’s usability and acceptability. We are also using evaluation tools including questionnaires measuring the same topics [68,69], and we are using the heiQ to identify psychosocial barriers to behavior change [62]. Another concern for some participants is that it is a time-consuming intervention because of its technology focus. Patients may choose not to participate in the study because of the time factor, and this may be a limitation to recruitment. It has been shown that recruitment to primary care trials is normally problematic, such as in the United Kingdom [69]. This may also be a problem in Norway, where there is less research in primary care than in specialist care. However, there is little research on how to improve recruitment in primary care studies. It is important to cooperate with health care personnel in the communities when developing an intervention directed toward “their” patients, and where user involvement is of great importance. Politicians and other stakeholders in the communities should both order and participate in research in this area [7]. This should help increase knowledge and competence for developing new and innovative services for health care users and providers.

## Abbreviations

EU: European Union
GP: general practitioner
HbA1c: hemoglobin A1c
heiQ: Health Education Impact Questionnaire
MAST: Model for Assessment of Telemedicine
MI: motivational interviewing
QALY: quality-adjusted life year
RENEWING HEALTH: REgioNs of Europe WorkINg together for HEALTH
SF-36: Short Form-36
SMS: short message service
SUS: System Usability Scale
T2D: type 2 diabetes
TM: telemedicine

## References

[1] Shaw JE, Sicree RA, Zimmet PZ (2010). Global estimates of the prevalence of diabetes for 2010 and 2030. Diabetes Res Clin Pract.

[2] Stene LC, Midthjell K, Jenum AK, Skeie S, Birkeland KI, Lund E, Joner G, Tell GS, Schirmer H (2004). [Prevalence of diabetes mellitus in Norway]. Tidsskr Nor Laegeforen.

[3] Hu FB (2011). Globalization of diabetes: the role of diet, lifestyle, and genes. Diabetes Care.

[4] Ali MK, Bullard KM, Saaddine JB, Cowie CC, Imperatore G, Gregg EW (2013). Achievement of goals in U.S. diabetes care, 1999-2010. N Engl J Med.

[5] Claudi T, Ingskog W, Cooper JG, Jenum AK, Hausken MF (2008). [Quality of diabetes care in Norwegian general practice]. Tidsskr Nor Laegeforen.

[6] UK Prospective Diabetes Study (UKPDS) Group (1998). Intensive blood-glucose control with sulphonylureas or insulin compared with conventional treatment and risk of complications in patients with type 2 diabetes (UKPDS 33). Lancet.

[7] Norwegian Ministry of HealthCare Services (2009). Report No. 47 (2008-2009) to the storting. The coordination reform. Proper treatment – at the right place and right time.

[8] Directorate for Health and Social Affairs (2009). National Guidelines. Diabetes. Prevention, Diagnosis and Treatment.

[9] Jenssen TG, Tonstad S, Claudi T, Midthjell K, Cooper J (2008). The gap between guidelines and practice in the treatment of type 2 diabetes: a nationwide survey in Norway. Diabetes Res Clin Pract.

[10] Cooper JG, Claudi T, Jenum AK, Thue G, Hausken MF, Ingskog W, Sandberg S (2009). Quality of care for patients with type 2 diabetes in primary care in Norway is improving: results of cross-sectional surveys of 33 general practices in 1995 and 2005. Diabetes Care.

[11] Norris SL, Nichols PJ, Caspersen CJ, Glasgow RE, Engelgau MM, Jack L, Snyder SR, Carande-Kulis VG, Isham G, Garfield S, Briss P, McCulloch D (2002). Increasing diabetes self-management education in community settings. A systematic review. Am J Prev Med.

[12] Tuomilehto J, Lindström J, Eriksson JG, Valle TT, Hämäläinen H, Ilanne-Parikka P, Keinänen-Kiukaanniemi S, Laakso M, Louheranta A, Rastas M, Salminen V, Uusitupa M, Finnish Diabetes Prevention Study Group (2001). Prevention of type 2 diabetes mellitus by changes in lifestyle among subjects with impaired glucose tolerance. N Engl J Med.

[13] Haas L, Maryniuk M, Beck J, Cox CE, Duker P, Edwards L, Fisher EB, Hanson L, Kent D, Kolb L, McLaughlin S, Orzeck E, Piette JD, Rhinehart AS, Rothman R, Sklaroff S, Tomky D, Youssef G, 2012 Standards Revision Task Force (2013). National standards for diabetes self-management education and support. Diabetes Care.

[14] Osborne RH, Batterham R, Livingston J (2011). The evaluation of chronic disease self-management support across settings: the international experience of the health education impact questionnaire quality monitoring system. Nurs Clin North Am.

[15] Norris SL, Lau J, Smith SJ, Schmid CH, Engelgau MM (2002). Self-management education for adults with type 2 diabetes: a meta-analysis of the effect on glycemic control. Diabetes Care.

[16] Ismail K, Thomas SM, Maissi E, Chalder T, Schmidt U, Bartlett J, Patel A, Dickens CM, Creed F, Treasure J (2008). Motivational enhancement therapy with and without cognitive behavior therapy to treat type 1 diabetes: a randomized trial. Ann Intern Med.

[17] Peyrot M, Rubin RR (2007). Behavioral and psychosocial interventions in diabetes: a conceptual review. Diabetes Care.

[18] Katon W (2010). Depression and diabetes: unhealthy bedfellows. Depress Anxiety.

[19] de Groot M, Kushnick M, Doyle T, Merrill J, McGlynn M, Shubrook J, Schwartz F (2010). Depression among adults with diabetes: prevalence, impact, and treatment options. Diabetes Spectr.

[20] Lin EH, Rutter CM, Katon W, Heckbert SR, Ciechanowski P, Oliver MM, Ludman EJ, Young BA, Williams LH, McCulloch DK, Von Korff M (2010). Depression and advanced complications of diabetes: a prospective cohort study. Diabetes Care.

[21] May CR, Finch TL, Cornford J, Exley C, Gately C, Kirk S, Jenkings KN, Osbourne J, Robinson AL, Rogers A, Wilson R, Mair FS (2011). Integrating telecare for chronic disease management in the community: what needs to be done?. BMC Health Serv Res.

[22] Castelnuovo G, Manzoni GM, Cuzziol P, Cesa GL, Corti S, Tuzzi C, Villa V, Liuzzi A, Petroni ML, Molinari E (2011). TECNOB Study: ad interim results of a randomized controlled trial of a multidisciplinary telecare intervention for obese patients with type-2 diabetes. Clin Pract Epidemiol Ment Health.

[23] Castelnuovo G, Manzoni GM, Cuzziol P, Cesa GL, Tuzzi C, Villa V, Liuzzi A, Petroni ML, Molinari E (2010). TECNOB: study design of a randomized controlled trial of a multidisciplinary telecare intervention for obese patients with type-2 diabetes. BMC Public Health.

[24] Verhoeven F, van Gemert-Pijnen L, Dijkstra K, Nijland N, Seydel E, Steehouder M (2007). The contribution of teleconsultation and videoconferencing to diabetes care: a systematic literature review. J Med Internet Res.

[25] Steventon A, Bardsley M, Billings J, Dixon J, Doll H, Hirani S, Cartwright M, Rixon L, Knapp M, Henderson C, Rogers A, Fitzpatrick R, Hendy J, Newman S, Whole System Demonstrator Evaluation Team (2012). Effect of telehealth on use of secondary care and mortality: findings from the Whole System Demonstrator cluster randomised trial. BMJ.

[26] Trief PM, Teresi JA, Eimicke JP, Shea S, Weinstock RS (2009). Improvement in diabetes self-efficacy and glycaemic control using telemedicine in a sample of older, ethnically diverse individuals who have diabetes: the IDEATel project. Age Ageing.

[27] Welschen LM, Bloemendal E, Nijpels G, Dekker JM, Heine RJ, Stalman WA, Bouter LM (2005). Self-monitoring of blood glucose in patients with type 2 diabetes who are not using insulin: a systematic review. Diabetes Care.

[28] Paré G, Moqadem K, Pineau G, St-Hilaire C (2010). Clinical effects of home telemonitoring in the context of diabetes, asthma, heart failure and hypertension: a systematic review. J Med Internet Res.

[29] Polisena J, Tran K, Cimon K, Hutton B, McGill S, Palmer K (2009). Home telehealth for diabetes management: a systematic review and meta-analysis. Diabetes Obes Metab.

[30] García-Lizana F, Sarría-Santamera A (2007). New technologies for chronic disease management and control: a systematic review. J Telemed Telecare.

[31] Chumbler NR, Neugaard B, Kobb R, Ryan P, Qin H, Joo Y (2005). Evaluation of a care coordination/home-telehealth program for veterans with diabetes: health services utilization and health-related quality of life. Eval Health Prof.

[32] Field MJ (1996). Telemedicine. A Guide to Assessing Telecommunications in Health Care.

[33] Chomutare T, Fernandez-Luque L, Arsand E, Hartvigsen G (2011). Features of mobile diabetes applications: review of the literature and analysis of current applications compared against evidence-based guidelines. J Med Internet Res.

[34] Farmer A, Gibson OJ, Tarassenko L, Neil A (2005). A systematic review of telemedicine interventions to support blood glucose self-monitoring in diabetes. Diabet Med.

[35] Ekeland AG, Bowes A, Flottorp S (2010). Effectiveness of telemedicine: a systematic review of reviews. Int J Med Inform.

[36] Shea S, Starren J, Weinstock RS, Knudson PE, Teresi J, Holmes D, Palmas W, Field L, Goland R, Tuck C, Hripcsak G, Capps L, Liss D (2002). Columbia University's Informatics for Diabetes Education and Telemedicine (IDEATel) Project: rationale and design. J Am Med Inform Assoc.

[37] Stone RA, Rao RH, Sevick MA, Cheng C, Hough LJ, Macpherson DS, Franko CM, Anglin RA, Obrosky DS, Derubertis FR (2010). Active care management supported by home telemonitoring in veterans with type 2 diabetes: the DiaTel randomized controlled trial. Diabetes Care.

[38] Nolte S, Elsworth GR, Newman S, Osborne RH (2012). Measurement issues in the evaluation of chronic disease self-management programs. Qual Life Res.

[39] Kidholm K, Ekeland AG, Jensen LK, Rasmussen J, Pedersen CD, Bowes A, Flottorp SA, Bech M (2012). A model for assessment of telemedicine applications: MAST. Int J Technol Assess Health Care.

[40] World Health Organization (2005). Preparing a Health Care Workforce for the 21st Century: The Challenge of Chronic Conditions.

[41] Richards D, Chellingsworth M, Hope R, Turpin T, Whyte M (2010). Reach Out: National Programme Supervisor Materials to Support the Delivery of Training for Psychological Wellbeing Practitioners Delivering Low Intensity Interventions.

[42] Richards D, Whyte M (2009). Reach Out: National Programme Educator Materials to Support the Delivery of Training for Psychological Wellbeing Practitioners Delivering Low Intensity Interventions.

[43] Rollnick S, Miller WR, Butler CC (2008). Motivational Interviewing in Health Care: Helping Patients Change Behavior (Applications of Motivational Interviewing).

[44] Prochaska JO, DiClemente CC, Norcross JC (1992). In search of how people change. Applications to addictive behaviors. Am Psychol.

[45] Prochaska JO, DiClemente CC (1983). Stages and processes of self-change of smoking: toward an integrative model of change. J Consult Clin Psychol.

[46] Vallis M, Ruggiero L, Greene G, Jones H, Zinman B, Rossi S, Edwards L, Rossi JS, Prochaska JO (2003). Stages of change for healthy eating in diabetes: relation to demographic, eating-related, health care utilization, and psychosocial factors. Diabetes Care.

[47] Walker EA, Shmukler C, Ullman R, Blanco E, Scollan-Koliopoulus M, Cohen HW (2011). Results of a successful telephonic intervention to improve diabetes control in urban adults: a randomized trial. Diabetes Care.

[48] Glasgow RE, Peeples M, Skovlund SE (2008). Where is the patient in diabetes performance measures? The case for including patient-centered and self-management measures. Diabetes Care.

[49] Sandelowski M (2000). Combining qualitative and quantitative sampling, data collection, and analysis techniques in mixed-method studies. Res Nurs Health.

[50] Årsand E (2009). The Few Touch Digital Diabetes Diary. User-Involved Design of Mobile Self-Help Tools for People with Diabetes.

[51] Årsand E, Tatara N, Østengen G, Hartvigsen G (2010). Mobile phone-based self-management tools for type 2 diabetes: the few touch application. J Diabetes Sci Technol.

[52] Craig P, Dieppe P, Macintyre S, Michie S, Nazareth I, Petticrew M, Medical Research Council Guidance (2008). Developing and evaluating complex interventions: the new Medical Research Council guidance. BMJ.

[53] Calvert M, Blazeby J, Altman DG, Revicki DA, Moher D, Brundage MD, CONSORT PRO Group (2013). Reporting of patient-reported outcomes in randomized trials: the CONSORT PRO extension. JAMA.

[54] Schulz KF, Altman DG, Moher D (2010). CONSORT 2010 statement: Updated guidelines for reporting parallel group randomised trials. J Pharmacol Pharmacother.

[55] NTNU The HUNT study - a longitudinal population health Study in Norway.

[56] McNamara R, Robling M, Hood K, Bennert K, Channon S, Cohen D, Crowne E, Hambly H, Hawthorne K, Longo M, Lowes L, Playle R, Rollnick S, Gregory JW (2010). Development and Evaluation of a Psychosocial Intervention for Children and Teenagers Experiencing Diabetes (DEPICTED): a protocol for a cluster randomised controlled trial of the effectiveness of a communication skills training programme for healthcare professionals working with young people with type 1 diabetes. BMC Health Serv Res.

[57] Wallace TM, Matthews DR (2000). Poor glycaemic control in type 2 diabetes: a conspiracy of disease, suboptimal therapy and attitude. QJM.

[58] Loge JH, Kaasa S (1998). Short Form 36 (SF-36) health survey: normative data from the general Norwegian population. Scand J Soc Med.

[59] Ware JE, Sherbourne CD (1992). The MOS 36-Item Short-Form Health Survey (SF-36). I. Conceptual framework and item selection. Med Care.

[60] Speight J, Reaney MD, Barnard KD (2009). Not all roads lead to Rome - a review of quality of life measurement in adults with diabetes. Diabet Med.

[61] Radloff L (1977). The CES-D Scale: A Self-Report Depression Scale for research in the general population. Appl Psychol Meas.

[62] Osborne RH, Elsworth GR, Whitfield K (2007). The Health Education Impact Questionnaire (heiQ): an outcomes and evaluation measure for patient education and self-management interventions for people with chronic conditions. Patient Educ Couns.

[63] Anderson RM, Fitzgerald JT, Gruppen LD, Funnell MM, Oh MS (2003). The Diabetes Empowerment Scale-Short Form (DES-SF). Diabetes Care.

[64] Anderson RM, Funnell MM, Fitzgerald JT, Marrero DG (2000). The Diabetes Empowerment Scale: a measure of psychosocial self-efficacy. Diabetes Care.

[65] Eigenmann CA, Colagiuri R, Skinner TC, Trevena L (2009). Are current psychometric tools suitable for measuring outcomes of diabetes education?. Diabet Med.

[66] Lorentzen C, Ommundsen Y, Holme I (2007). Psychosocial correlates of stages of change in physical activity in an adult community sample. Eur J Sport Sci.

[67] Larsen IK, Grotmol T, Almendingen K, Hoff G (2006). Lifestyle characteristics among participants in a Norwegian colorectal cancer screening trial. Eur J Cancer Prev.

[68] Brooke J, Jordan PW, Thomas B, Weerdmeester BA, McClelland AL (1996). SUS: a "quick and dirty" usability scale. Usability Evaluation in Industry.

[69] Bower P, Cartwright M, Hirani SP, Barlow J, Hendy J, Knapp M, Henderson C, Rogers A, Sanders C, Bardsley M, Steventon A, Fitzpatrick R, Doll H, Newman S (2011). A comprehensive evaluation of the impact of telemonitoring in patients with long-term conditions and social care needs: protocol for the whole systems demonstrator cluster randomised trial. BMC Health Serv Res.

[70] Strauss AL, Corbin JM (1998). Basics of Qualitative Research: Techniques and Procedures for Developing Grounded Theory.

[71] Brazier J, Roberts J, Deverill M (2002). The estimation of a preference-based measure of health from the SF-36. J Health Econ.

[72] Drummond MF, McGuire A (2001). Economic Evaluation in Health Care: Merging Theory with Practice.

[73] Bradley C (1993). Designing medical and educational intervention studies. A review of some alternatives to conventional randomized controlled trials. Diabetes Care.

[74] Bradley C (1996). Patients' preferences and randomised trials. Lancet.

[75] Harwell TS, Dettori N, McDowall JM, Quesenberry K, Priest L, Butcher MK, Flook BN, Helgerson SD, Gohdes D (2002). Do persons with diabetes know their (A1C) number?. Diabetes Educ.

[76] Nolte S, Osborne RH (2012). A systematic review of outcomes of chronic disease self-management interventions. Qual Life Res.

[77] Polit DF, Beck CT (2008). Nursing Research: Generating and Assessing Evidence for Nursing Practice. 8th edition.

[78] Glasgow RE, Hiss RG, Anderson RM, Friedman NM, Hayward RA, Marrero DG, Taylor CB, Vinicor F (2001). Report of the health care delivery work group: behavioral research related to the establishment of a chronic disease model for diabetes care. Diabetes Care.

[79] Bower P, Wallace P, Ward E, Graffy J, Miller J, Delaney B, Kinmonth AL (2009). Improving recruitment to health research in primary care. Fam Pract.

[80] Sanders C, Rogers A, Bowen R, Bower P, Hirani S, Cartwright M, Fitzpatrick R, Knapp M, Barlow J, Hendy J, Chrysanthaki T, Bardsley M, Newman SP (2012). Exploring barriers to participation and adoption of telehealth and telecare within the Whole System Demonstrator trial: a qualitative study. BMC Health Serv Res.

